# Volunteering as a generative act and contribution to a good life in older age: support by general practice through social prescribing – a qualitative focus group study protocol in Germany

**DOI:** 10.1136/bmjopen-2026-126407

**Published:** 2026-10-26

**Authors:** Evelyn Kleinert, Laura Mohacsi, Eva Hummers

**Affiliations:** 1 Department of General Practice, University Medical Center Göttingen, Göttingen, Germany

**Keywords:** Primary Care, QUALITATIVE RESEARCH, SOCIAL MEDICINE, Quality of Life, Aging, Community Participation

## Abstract

**Introduction:**

Loneliness and social isolation in older age are increasingly recognised as major public health concerns, associated with depression, cognitive decline, cardiovascular disease and premature mortality. Despite this recognition, primary care lacks systematic approaches to address the social determinants of health in ageing populations. Social prescribing (SP)—the structured referral of patients to non-clinical, community-based activities by primary care professionals—has been proposed as a promising response to this gap. While SP is well established in the UK and increasingly implemented internationally, it remains largely unexplored in the German healthcare context. This study examines how volunteering as a form of generativity contributes to health and a good life in older age, and how general practice can support this through SP.

**Methods and analysis:**

A qualitative focus group design is employed, involving three participant populations: (1) current and former volunteers aged 60 and older, (2) younger adults unable to work due to chronic illness and (3) interprofessional groups of general practitioners, social workers and local association representatives. Recruitment is multimodal to ensure diversity of background and experience. All sessions are audio-recorded and analysed using qualitative content analysis according to Kuckartz.

**Ethics and dissemination:**

Ethical approval has been granted by the Ethics Committee of the University Medical Center Göttingen (Ethics Approval No. 22/8/25). All participants will provide written informed consent prior to participation; data will be pseudonymised and processed in accordance with the General Data Protection Regulation (GDPR). Findings will be disseminated through peer-reviewed publication and presented at national and international conferences in the fields of general practice and public health. A practice-oriented summary will be made available to participants and relevant stakeholders to support the translation of findings into primary care practice.

**Trial registration number:**

DRKS00037936.

**STRENGTHS AND LIMITATIONS OF THIS STUDY**

- This study employs a multigroup qualitative focus group design that systematically integrates the perspectives of older volunteers, chronically ill younger adults and interprofessional stakeholders, enabling a comparative and theoretically grounded analysis that goes beyond single-population approaches.
- The study builds on an established German Research Foundation-funded research programme with an approved methodological framework and prior qualitative evidence from a similar population, strengthening the credibility and continuity of the research design.
- The involvement of a Citizen Advisory Board at multiple stages enhances the participatory quality and accessibility of the study.
- As a qualitative study, findings will not be statistically generalisable; transferability to other primary care contexts—particularly outside the German healthcare system—will depend on the degree of structural similarity between settings.
- Recruitment through general practices and voluntary organisations may introduce selection bias, as participants who are already engaged with these structures may differ systematically from those who are more socially isolated and therefore most at risk.

## Introduction

 Worldwide, loneliness and social isolation in older age have become central public health challenges of the 21st century. Up to one-third of older European adults report feeling lonely,^1^ and the health consequences are substantial: loneliness is associated with increased risk of depressive symptoms, cognitive decline and dementia progression, hypertension, heart disease, stroke and premature mortality.^2–5^ The relationship between loneliness and mental health is particularly well established, with evidence of a reciprocal dynamic in which loneliness and depressive symptoms mutually reinforce one another.^6 7^ Older women who are widowed, divorced or single, have few close friendships, experience chronic illness or have limited socioeconomic resources are at particular risk.^8^ Life transitions such as retirement or bereavement further amplify this vulnerability. In Germany, the scale of the problem has prompted a national strategy against loneliness by the federal government,^9^ yet systematic approaches within general practice to address social isolation remain absent.^10^

Social participation and volunteering in older age offer a promising, theoretically grounded response to these challenges. Antonovsky’s salutogenesis model identifies meaningfulness—alongside manageability and comprehensibility—as a fundamental determinant of health and well-being.^11^ Erikson’s concept of generativity, broadly interpreted by King as the intergenerational transmission of knowledge, care and resources independent of biological reproduction, provides a compelling framework for understanding how older adults experience and derive meaning from social roles.^12^ Kruse refers to this phenomenon as ‘co-responsible’ living in older age: older individuals are not merely recipients of care, but actively contribute to others’ well-being.^13^ Volunteering and community engagement can thus be understood as generative acts that sustain a sense of purpose and social connectedness simultaneously—and that may meaningfully buffer against the health consequences of loneliness and social isolation. Despite this theoretical grounding, the conditions under which general practice can actively support such participation remain empirically underexplored, particularly in non-Anglophone healthcare contexts.

Social prescribing (SP) represents a structural approach through which primary care professionals can actively support such participation. Based on a biopsychosocial model of health, SP involves the referral of patients by general practitioners, nurses or social workers to non-pharmacological, community-based activities tailored to individual needs—ranging from physical exercise and creative activities to volunteering. In the UK, SP is now comprehensively embedded in National Health Service primary care, and international implementation is expanding rapidly.^14^ In Germany, by contrast, SP remains at an early stage of discussion, and primary care providers currently lack both the frameworks and the evidence base to identify suitable patients or to navigate existing community structures.^15^ This absence is particularly significant given that general practitioners are frequently among the first to recognise social risk factors such as loneliness, yet have no systematic tools to address them.^16^

The present study is embedded in an established German Research Foundation (DFG)-funded research programme (FOR 5022 ‘Medicine, Time and the Good Life’), which has generated foundational qualitative evidence on ageing, health and social participation in Germany.^17–20^ The study protocol for the first funding period was published in BioMed Central (BMC) Health Services Research,^21^ providing the methodological foundation on which the current protocol builds. In the first funding period, focus groups with adults aged 75 and above and their care providers identified meaningful social participation and volunteering as central components of a good life in older age.^22 23^ Participants described interpersonal relationships and integration into social networks as an essential foundation for well-being, and proposed building such networks proactively—‘while one is still well’—as a strategy against the fear of future isolation. They also criticised the frequent pharmacological treatment of age-related ailments, arguing that some prescriptions could be avoided through supportive social relationships and meaningful activity. These findings now raise a question to be addressed in the second funding period: how can primary care systematically support such participation, and can SP provide a viable framework for doing so in the German context?

The present study advances this work in two key respects. First, it extends the participant population to include adults aged 60 and older, including the cohort born between 1955 and 1965. Many of them are entering or approaching retirement, a life transition that can disrupt established routines and trigger social withdrawal. Second, it introduces interprofessional focus groups with general practitioners, social workers and civil society representatives, enabling the perspectives of potential SP implementers to be examined alongside those of patients and community members. The findings are intended to generate practical guidance for identifying and supporting appropriate patient groups, and to inform the adaptation of SP to the structural conditions of the German healthcare system.

Since the qualitative design is hypothesis-generating rather than hypothesis-testing, research questions are formulated as open and provisional, to be continuously refined during the study. The study addresses four inter-related questions: (1) How do individuals perceive the relationship between volunteering, social participation and their own health and well-being? (2) What role does social participation play as a coping strategy when the structuring and meaning-providing function of employment is lost, through retirement or chronic illness? (3) How do generative motivations—the desire to transmit knowledge, care and time to others—shape decisions to take on social roles in later life? (4) How can SP be embedded in general practice in Germany; what opportunities and barriers are perceived by different professional groups; and how do interprofessional networks shape the implementation process?

## Methods

### Design

The project will build on the focus group methodology successfully employed in the first funding period, as the research questions retain an exploratory character and rely on narratives and patterns of argumentation. The effect described in the literature for patient groups—that focus groups foster authentic statements and increase willingness to speak openly about sensitive topics among peers^24^—was also observed in our groups with older adults during the first funding period and is expected to apply equally in the present study. Each focus group session is structured around a semiopen guide developed iteratively by the research team. To lower the threshold for personal disclosure and stimulate narrative exchange, the guide incorporates short thematic case vignettes depicting fictional older adults in situations relevant to the study’s research questions—including transitions such as retirement, chronic illness and the decision to take on or relinquish a voluntary role. This approach draws on established practice in qualitative focus group research, in which fictional scenarios serve as projective stimulus material, enabling participants to engage with potentially sensitive topics at a degree of distance from their own biography.^25^ The focus group guides, including all case vignettes, are provided as online supplemental file 1.

Four participant groups are planned, as detailed in table 1: current volunteers aged 60 and older (BeG1), former volunteers aged 60 and older (BeG2), younger adults unable to work due to chronic illness (BeG3), and interprofessional groups of general practitioners, social workers and civil society representatives (FachG). Groups BeG1–3 will meet for two group discussion sessions with the same participants, each lasting 90–120 min. This expanded format is intended to foster greater familiarity within the group dynamic and facilitate a more in-depth discussion.^26^ The interprofessional groups (FachG) will be conducted in the classic single-session format of 90–120 min, given participants’ limited availability and presumed prior knowledge. Experience from the first funding period indicates that focus groups with professionals yield more concise knowledge-based contributions rather than personal narratives. All focus groups will be audio-recorded.

**Table 1:** Overview of the planned focus groups

Abbreviation	Focus group participants	Number of focus groups	Number of participants	Type of focus group
BeG1	Volunteers aged 60 and above	3–4	15–24	Extended*
BeG2	Former volunteers aged 60 and above	1–2	5–12	Extended*
BeG3	Individuals unable to work due to chronic illness	1–2	5–12	Extended*
FachG	Interprofessional groups of general practitioners, social workers and representatives of local associations	3	15–18	Classic†

* Extended focus group: two sessions of 90–120 min each with the same group composition.

† Classic focus group: one session per group of 90–120 min.

Qualitative focus group research does not lend itself to statistical power calculations; instead, sample size is justified on the basis of theoretical saturation—the principle that data collection continues until no new themes or categories emerge from additional groups.^27^ The exact number of groups is not fixed in advance but will be determined iteratively during the analytical process, consistent with established guidance recommending a minimum of three to six groups per population stratum to achieve thematic saturation.^28^ Based on the planned group compositions detailed in table 1, an estimated total of 40 to 66 participants will be recruited across all strata. This range is consistent with the experience gained in the first funding period of the same research programme, in which thematic saturation was reliably achieved within a comparable sample^21^ and accords with the concept of information power as a basis for sample size decisions in qualitative research.^26^ Data collection will take place from March 2026 to July 2027.

### Recruitment/sampling

Recruitment of volunteer and former volunteers aged 60 and above (BeG1 and BeG2) will be conducted not only through contacts with general practices but also via press releases to local daily and free newspapers, which reach broad segments of the population, as well as through social media channels commonly used by this target group. In addition, local organisations and volunteer placement agencies will serve as cooperation partners for reaching volunteers. For the recruitment of groups with chronically ill individuals who are unable to work (BeG3), an existing connection to the Göttingen Contact Point for Self-Help Groups (KIBIS) and established networks within general practice will be used. Other potential cooperation partners include the pension insurance fund and the supplementary independent participation counselling service for people with disabilities (EUTB).

To reflect the diversity of sociocultural characteristics within each participant group, selection for groups BeG1–3 will consider not only age and gender but also educational background, cultural background, health status and any disabilities. To enable participation by individuals with physical impairments, group discussions will be held in accessible, barrier-free locations. During the first funding period, people with a migration background were rarely willing to participate in the study—even when specifically approached through relevant organisations and directly by cooperating general practitioners. Therefore, two medical doctoral candidates with native language skills and personal migration experience will be part of the research team. Their role is to assist with the recruitment of participants from migrant communities by translating study materials and, if necessary, enabling participants to communicate in their native language. To minimise interpretation bias, all translated contributions will be audio-recorded alongside the original utterances, enabling post hoc verification by the research team where possible. Personnel will be briefed to provide verbatim translation as possible rather than summary interpretation. Any uncertainty about translation choices will be discussed with the entire team after the focus group sessions.

General practitioners will again be recruited through existing research and teaching contacts of the Department of General Practice at the University Medical Center Göttingen (UMG), which were successfully used for data collection in the first funding period. Social workers and representatives of local civil society organisations can also be approached in part through existing contacts.

### Inclusion and exclusion criteria

Inclusion criteria for group BeG1:

- Age 60 or older.
- Existing social participation or volunteering.

Inclusion criteria for group BeG2:

- Age 60 or older.
- Previous social participation or volunteering.

Inclusion criteria for group BeG3:

- Age 18 or older.
- Existing reduction in earning capacity due to chronic illness.
- Existing social participation or volunteering.

Inclusion criteria for group FachG:

- Professional activity as a general practitioner, social worker or in local associations and civil society structures.

General inclusion criteria (apply to all four groups):

- Sufficient language skills.
- Interest in the research question.
- Willingness to participate in the study and consent to audio recording of the discussions.

General exclusion criteria (apply to all four groups):

- Insufficient language skills.
- Lack of cognitive and social ability to participate in a structured group discussion.

### Withdrawal and discontinuation criteria

Data collection and analysis will be discontinued immediately if a participant withdraws consent to participate in the study. As the study involves only interviews and no interventions, no additional discontinuation criteria are required.

### Data analysis

Data analysis follows a five-phase process based on qualitative content analysis according to Kuckartz,^29^ as illustrated in figure 1, and supported by MAXQDA software. All focus group sessions are audio-recorded, transcribed verbatim and pseudonymised prior to analysis. Inductive categories will be derived from the data, while the concepts of quality of life, SP and generativity will serve as deductive starting categories, to be validated and expanded through in-depth analysis. Given the active involvement of researchers in all focus group sessions, analytical memo writing and initial theme identification begin during data collection itself. For systematic coding, selected transcripts are coded independently in parallel by at least two researchers to establish a shared coding frame through consensus; remaining transcripts are then distributed among team members and discussed in regular team meetings. Data collection and analysis proceed iteratively and in parallel: after each focus group session, emerging themes are reviewed by the research team using the constant comparative method, informing decisions about whether further data collection is required to achieve thematic saturation. Once saturation is reached, data collection ceases and the final synthesis phase begins, resulting in a typology of patient backgrounds, motivations and barriers to inform a practical guide for general practitioners implementing SP.

**Figure 1:**
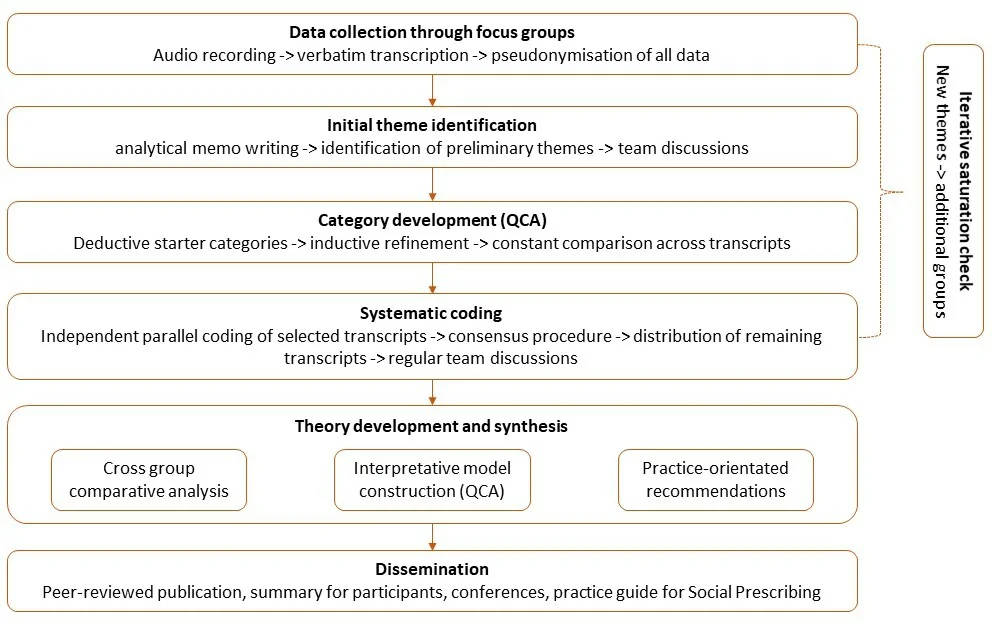
Data analysis plan. QCA, qualitative content analysis.

To describe the sample and ensure maximum diversity, the following sociodemographic variables will be collected and analysed descriptively: age, gender, migration background, highest educational attainment; for professionals (FachG): field of specialisation, institutional affiliation, years of work experience and experience with SP; for BeG1–3: years until/since retirement or work disability, household type, self-assessed health status, financial concerns and type of volunteering activity.

To ensure the trustworthiness of findings, this study explicitly addresses the four criteria proposed by Lincoln and Guba^30^: credibility is supported through independent parallel coding, consensus procedures and analytical memo writing, as described above. Confirmability is ensured through a reflexive audit trail documenting all analytical decisions throughout the study. Dependability is addressed through the iterative, transparent parallel process of data collection and analysis. Transferability will be facilitated through thick contextual description of the study setting and participant characteristics, as outlined in the Discussion. As a form of communicative validation, the moderator will briefly summarise the key themes at the end of each focus group session and invite participants to confirm, correct or expand on the summary.

### Patient and public involvement

Members of the public were not formally involved in the initial design of the present study prior to its initiation. However, the research questions and thematic focus of this second funding period were directly informed by findings from the first funding period of the same DFG-funded research programme (FOR 5022), in which older adults aged 75 and above participated in qualitative focus groups. Their accounts—particularly their emphasis on the health-protective role of social participation and volunteering, and their criticism of purely pharmacological responses to age-related social challenges—substantively shaped the conceptual framework and research questions of the present study. In this sense, public perspectives were integral to the iterative development of the research agenda through prior empirical work.

For the present study, structured public involvement is implemented through the Citizen Advisory Board of the Northern German Network of Research Practices (FoPraNet-Nord).^31^ The Board, which includes lay members with diverse biographical and health-related backgrounds, is involved at several stages of the research process: in the planning and design of the focus group sessions, in the development of participant information and informed consent materials, and in the subsequent preparation of findings in a format accessible to non-academic audiences. Four to five advisory meetings are planned for this purpose over the course of the study. This involvement is implemented at an advisory level.^32^ Within the study itself, participants are further involved as co-producers of knowledge through the focus group design.

Findings will be disseminated not only through academic publication and conference presentation, but also through lay-oriented summaries developed in collaboration with the Citizen Advisory Board and made available to participating community organisations, voluntary associations and general practices.

## Ethics and dissemination

###  Ethical approval

The study will be conducted in accordance with the current version of the Declaration of Helsinki and the principles of good scientific practice. The study protocol was approved by the Ethics Committee of the University Medical Center Göttingen prior to the commencement of the study (Ethics Approval No. 22/8/25). Data collection will comply with the General Data Protection Regulation of the European Union (GDPR), the Federal Data Protection Act (BDSG), and the federal, health, and state data protection laws of Lower Saxony.

Study participation is voluntary, and consent can be withdrawn at any time without providing reasons and without any adverse consequences. Before participation, all individuals will receive written information outlining the nature, scope and purpose of the study. Consent will be documented by a signed declaration of participation. In the event of withdrawal, all personal data will be deleted immediately; focus group data already collected will be anonymised and may continue to be used in anonymised form.

### Dissemination

Findings will be disseminated through peer-reviewed publication and presented at national and international conferences in the fields of general practice, gerontology and public health. A practice-oriented summary will be made available to participants, participating general practices and relevant community organisations to support the translation of findings into primary care practice. Lay-oriented summaries will be developed in collaboration with the Citizen Advisory Board of FoPraNet-Nord and shared with voluntary associations and civil society partners involved in the study.

Anonymised transcripts and contextual information will be stored in password-protected form at the Department of General Practice, University Medical Center Göttingen, for a period of 10 years following completion of data analysis, in accordance with the data management plan approved by the DFG.

## Discussion

This study protocol describes a qualitative focus group design to examine how volunteering as a generative act contributes to health and a good life in older age, and how general practice can support this through SP. To our knowledge, it is the first protocol in the German context to integrate generativity, social participation and SP as a triadic framework for health-promoting primary care—addressing a gap that is both theoretically well-founded and practically urgent.

The study builds on robust evidence that social participation and meaningful activity buffer against the health consequences of loneliness and social isolation in older age. By extending this evidence base to include the perspective of primary care professionals and civil society players, the study moves beyond documenting individual experience towards generating actionable guidance for system-level implementation. The interprofessional focus group design is particularly suited to this aim. Bringing together general practitioners, social workers and community representatives enables the negotiation of shared frameworks and the identification of collaborative pathways at a stage when SP has not yet been formally implemented in Germany

The inclusion of a contrast group of younger adults unable to work due to chronic illness (BeG3) adds comparative depth that goes beyond most existing SP research, which tends to focus exclusively on older populations. This group faces structurally similar challenges—loss of daily routine, reduced social integration and diminished sense of purpose—but in a different biographical context, enabling the study to examine whether the generativity framework travels across life phases and health conditions.

### Anticipated limitations

Several limitations should be acknowledged. First, as a qualitative study, findings will not be statistically generalisable; transferability to other primary care contexts will depend on the degree of structural similarity between settings and on the richness of the contextual description provided. Second, recruitment through general practices and voluntary organisations may introduce selection bias: participants already engaged with these structures may differ systematically from those who are most socially isolated and therefore most in need of SP. Third, the focus group format, while well suited to generating shared narratives and group-level dynamics, may suppress minority views or discourage disclosure of stigmatised experiences—a risk that the use of case vignettes is designed to partially mitigate. Fourth, the interprofessional composition of the FachG groups may create professional hierarchies within sessions that affect the openness of discussion; this will be actively managed through moderation.

### Implications and transferability

Germany represents an instructive case for international SP implementation research: a highly developed primary care system with well-established gatekeeping functions, but without the Link Worker infrastructure that underpins SP in the UK. The practical guide to be developed from this study’s findings is therefore designed not only for the German context but as a transferable model for healthcare systems at an early stage of SP adoption. As SP continues to expand globally, qualitative evidence on implementation barriers, motivational dynamics and interprofessional collaboration from non-Anglophone contexts will be increasingly important for informing context-sensitive adaptation. This study is intended as a contribution to that evidence base.

## Supplementary material

10.1136/bmjopen-2026-126407 online supplemental file 1
