# Supplementary material for: Volunteering as a generative act and contribution to a good life in older age: support by general practice through social prescribing – a qualitative focus group study protocol in Germany
Source: BMJ Open. 2026 Oct 26;16(10):e126407. doi: 10.1136/bmjopen-2026-126407 (PMC13711605; doi:10.1136/bmjopen-2026-126407)
Supplement: Supplementary file 1 [file bmjopen-2026-126407.reviewer_comments.pdf]

PEER REVIEW HISTORY

BMJ Open publishes all reviews undertaken for accepted manuscripts. Reviewers are asked to complete a checklist review form and are provided with free text boxes to elaborate on their assessment. These free text comments are reproduced below.

ARTICLE DETAILS

Title (Provisional)

Volunteering as a generative act and contribution to a good life in older age -support by general practice through Social Prescribing: a qualitative focus group study protocol in Germany

Authors

Kleinert, Evelyn; Mohacsi, Laura; Hummers, Eva

VERSION 1 - REVIEW

**Reviewer**	**1**
**Name**	**Ramsden, Vivian**
**Affiliation**	**University of Saskatchewan, Department of Academic Family Medicine**
**Date**	**09-Sep-2026**
**COI**	**none**

The Investigators indicated that they were planning a qualitative focus group study protocol which would consider ways to address social determinants of health in aging populations. The Investigators went on to consider how “social prescribing” which is a structured referral of patients to non-clinical, community-based activities by primary care professionals which was identified as a promising response for bridging the gap specifically in the UK. Thus, they broke the focus groups down into three “participant” populations: 1. Current and former volunteers aged 60 years and older (two groups); 2. Younger adults unable to work due to chronic illness (one group); and 3. Interprofessional groups of general practitioners, social workers and local association representatives (one group).

Given that this is a Protocol, it would be helpful for the Investigators to specifically frame how the planned qualitative focus groups will address patient and public involvement. From my reading of this protocol, patient and public involvement consisted of participant responses rather than either involvement or engagement. This being said, the Investigators chose extended focus groups for groups made up of populations outlined in groups 1, 2 and 3. It would appear that this was to in some way support build relationships, but it impacts the outcomes and does not help with keeping the focus groups the same.

Given this is a protocol, the language should reflect either future or being in the planning stages rather than past tense as if it has been completed. The Investigators indicated that they

would recruit two medical doctoral candidates with personal migration experience as "cultural guides". This is a significant power imbalance and may impact participants in groups 1, 2 and 3.

The analysis planned will be a qualitative content analysis with inductive categories being derived from the data, and these concepts will serve as deductive starting categories. All of this may be possible but then the Investigators indicate that they will supplement this data with aspects of Grounded Theory. Although these are two different methods within qualitative research methodologies, it would appear that the Investigators are using Grounded Theory which then needs to be written using that language e.g. constant comparative method, memo writing and theoretical saturation. It will also be important for the Investigators to address all aspects of qualitative rigour (credibility, confirmability, dependability and transferability along with member-checking and spending time in the community) as these all contribute to the success of any qualitative study. However, if the Investigators are going to use two different qualitative methods, it will be very important for them to describe how these two approaches will be integrated in such a way that qualitative researchers find the findings or theory developed credible.

This protocol could have benefitted from having patient partners/people with lived and living experience involved in the co-creation of the research project in addition to a Citizen Advisory Board.

VERSION 1 - AUTHOR RESPONSE

Review 1 with Point-by-Point response

The Investigators indicated that they were planning a qualitative focus group study protocol which would consider ways to address social determinants of health in aging populations. The Investigators went on to consider how "social prescribing" which is a structured referral of patients to non-clinical, community-based activities by primary care professionals which was identified as a promising response for bridging the gap specifically in the UK. Thus, they broke the focus groups down into three "participant" populations: 1. Current and former volunteers aged 60 years and older (two groups); 2. Younger adults unable to work due to chronic illness (one group); and 3. Interprofessional groups of general practitioners, social workers and local association representatives (one group).

Given that this is a Protocol, it would be helpful for the Investigators to specifically frame how the planned qualitative focus groups will address patient and public involvement. From my reading of this protocol, patient and public involvement consisted of participant responses rather than either involvement or engagement.

> We thank the reviewer for this comment. We would like to clarify that the Patient and Public Involvement section already describes in detail the nature and scope of public involvement in this study, including an explicit acknowledgement of its limitations. The Citizen Advisory Board of FoPraNet-Nord is involved at an advisory level in the planning of focus group sessions, the development of participant information and consent materials, and the preparation of lay-oriented summaries of findings. We have been intentionally transparent that this involvement does not constitute formal co-creation of the research project, as the study was not designed as an explicit PPI project. We believe that overstating the degree of

PPI would misrepresent the actual design of the study. In addition, we wish to reiterate that the research questions and conceptual framework of the present study were substantively shaped by the findings and perspectives of older adult participants in the first funding period — a form of indirect public involvement that is explicitly described in the section. We consider the current PPI section to accurately and transparently reflect the participatory elements of this study and have retained it largely unchanged.

This being said, the Investigators chose extended focus groups for groups made up of populations outlined in groups 1, 2 and 3. It would appear that this was to in some way support build relationships, but it impacts the outcomes and does not help with keeping the focus groups the same.

The extension involves the groups meeting on two consecutive dates with the same members. We have rephrased the sentence as follows to make this clearer.

"Groups BeG1–3 will meet for two group discussion sessions with the same participants, each lasting 90–120 minutes. This expanded format is intended to foster greater familiarity within the group dynamic and facilitate a more in-depth discussion. [26]"

Given this is a protocol, the language should reflect either future or being in the planning stages rather than past tense as if it has been completed.

We have carefully reviewed the manuscript for tense consistency. Two passages use past tense legitimately, as they refer explicitly to completed work from the first funding period that provides the empirical and methodological foundation for the current study. We have added a brief clarifying phrase on page 4 to make this reference unambiguous and prevent any confusion with the current study's ongoing data collection.

"The effect described in the literature for patient groups – that focus groups foster authentic statements and increase willingness to speak openly about sensitive topics among peers [24] – was also observed in our groups with older adults during the first funding period and is expected to apply equally in the present study."

The text on p. 6 explicitly refers to the first funding phase, which is why the use of the past tense is justified.

"During the first funding period, people with a migration background were rarely willing to participate in the study – even when specifically approached through relevant organizations and directly by cooperating general practitioners."

The Investigators indicated that they would recruit two medical doctoral candidates with personal migration experience as "cultural guides". This is a significant power imbalance and may impact participants in groups 1, 2 and 3.

We thank the reviewer for raising the concern about power imbalance. On reflection, we understand this to refer in part to the risk of interpreter or translation bias: cultural guides who translate participant contributions may inadvertently select, smooth, or interpret rather than render verbatim, potentially distorting the data without the research team's

awareness. We have added the following paragraph to the Recruitment section at page 7 to address this point:

"To minimise interpretation bias, all translated contributions will be audio-recorded alongside the original utterances, enabling post-hoc verification by the research team where possible. Personnel will be briefed to provide verbatim translation as possible rather than summary interpretation. Any uncertainty about translation choices will be discussed with the entire team after the focus group sessions."

The analysis planned will be a qualitative content analysis with inductive categories being derived from the data, and these concepts will serve as deductive starting categories. All of this may be possible but then the Investigators indicate that they will supplement this data with aspects of Grounded Theory. Although these are two different methods within qualitative research methodologies, it would appear that the Investigators are using Grounded Theory which then needs to be written using that language e.g. constant comparative method, memo writing and theoretical saturation.

This is an important methodological observation. On reflection, we agree that the reference to Grounded Theory introduced unnecessary methodological complexity and potential inconsistency. We have therefore removed all references to Grounded Theory from the manuscript. The iterative, parallel process of data collection and analysis — in which emerging themes are reviewed after each focus group session to inform decisions about further data collection and thematic saturation — is now described as standard practice in qualitative research without invoking a specific GT framework. QCA according to Kuckartz remains the sole analytical method. We believe this revision strengthens the methodological coherence of the protocol.

It will also be important for the Investigators to address all aspects of qualitative rigour (credibility, confirmability, dependability and transferability along with member-checking and spending time in the community) as these all contribute to the success of any qualitative study. However, if the Investigators are going to use two different qualitative methods, it will be very important for them to describe how these two approaches will be integrated in such a way that qualitative researchers find the findings or theory developed credible.

We thank the reviewer for drawing attention to the absence of explicit quality criteria. We agree that the trustworthiness framework should be more visibly addressed. We wish to note, however, that several of the criteria outlined by Lincoln and Guba were already implicitly present in the manuscript — specifically, the reflexive audit trail (confirmability), the parallel coding and consensus procedure (credibility), and the reference to thick description (transferability). To make this more explicit, we added the following paragraph to the "Methods" section (page 6):

"To ensure the trustworthiness of findings, this study explicitly addresses the four criteria proposed by Lincoln and Guba [30]: credibility is supported through independent parallel coding, consensus procedures, and analytical memo writing, as described above. Confirmability is ensured through a reflexive audit trail documenting all analytical decisions throughout the study. Dependability is addressed through the iterative, transparent parallel process of data collection and analysis. Transferability will be facilitated through thick contextual description of the study setting and participant characteristics, as outlined in the Discussion. As a form of communicative validation, the moderator will briefly summarise the

key themes at the end of each focus group session and invite participants to confirm, correct, or expand upon the summary."

The suggestion to spend time within the community—as an important component of ethnographic research—is not a feature of the research design of this study, which is based on a targeted qualitative focus group method. Therefore, this aspect is not included in the present study protocol.

This protocol could have benefitted from having patient partners/people with lived and living experience involved in the co-creation of the research project in addition to a Citizen Advisory Board.

We thank the reviewer for this comment. We acknowledge that formal co-creation of the research project with patient partners or people with lived and living experience was not implemented prior to the initiation of this study. However, we would like to draw the reviewer's attention to the Patient and Public Involvement section of the manuscript, which describes in detail the participatory elements that are in place. The Citizen Advisory Board (CAB) of FoPraNet-Nord, which includes lay members with diverse biographical and health-related backgrounds, is actively involved at multiple stages of the research process, including the planning and design of focus group sessions, the development of participant information and consent materials, and the preparation of findings for non-academic audiences. Furthermore, the research questions and conceptual framework of the present study were substantively shaped by the accounts and priorities of older adult participants in the first funding period — a form of indirect but meaningful public involvement through prior empirical engagement. We have been intentionally transparent in the manuscript that this involvement is implemented at an advisory level and does not constitute formal co-creation. We believe this accurately reflects the participatory approach of this study and that overstating the degree of PPI would misrepresent the actual design. The reviewer's suggestion is noted as a valuable recommendation for future research in this programme.
