# Supplementary material for: Volunteering as a generative act and contribution to a good life in older age: support by general practice through social prescribing – a qualitative focus group study protocol in Germany
Source: BMJ Open. 2026 Oct 26;16(10):e126407. doi: 10.1136/bmjopen-2026-126407 (PMC13711605; doi:10.1136/bmjopen-2026-126407)
Supplement: Supplementary file 2 [file bmjopen-16-10-s001.docx]

# **Focus Group Guide — BeG 1–3 (part 1)**

*Group-specific adaptations are indicated in brackets [BeG1 / BeG2 / BeG3]. All other content applies equally to all three groups.*

| Introduction and Project Overview:  This project is a follow-up to our previous project, “Medicine in old age.” During the first funding period, we also conducted focus group discussions with doctors, nurses, and people over the age of 75. Among other things, these discussions revealed that social participation and commitment are important factors for well-being and health in later life. Staying socially active, feeling connected, and maintaining physical and mental fitness were repeatedly emphasized as a key to a good quality of life in old age. Volunteering was consistently cited as a good way to achieve this. We are now addressing these aspects in the second funding period. That is why we have invited you here today—to talk to you about your voluntary activity and learn a little more about it from you. (10 min) | | | |
| --- | --- | --- | --- |
| Narrative prompt | **Check // Memos** | **Specific questions** | **Directive and maintaining questions** |
| Case vignette 1.1  (20–25 min) | Which motivators?  Which barriers? | What do you think about this example in light of your own experiences? | Do you recognise aspects of this from your own experience?  What should Achim [the person in the vignette] do now?  What would you do in their position? |
| Case vignette 1.2  (20–25 min)  *(Can be omitted if the first case vignette generates enough relevant narratives)* | Which motivators?  Which barriers? | What do you think about this example in light of your own experiences? | Do you recognise aspects of this from your own experience?  What should Anna [the person in the vignette] do now?  What would you do in their position? |
| [BeG1 & BeG3] Please tell us about your current voluntary activity.  [BeG2] Please tell us about your past voluntary activity — or any plans you may have to volunteer again.  (35–40 min) | Original motivation  Sustaining voluntary activity  Difficulties and coping  Expectation vs. reality | How did you come to [take on / take on and leave / develop] this activity?  What have you tried in order to overcome any difficulties?  [BeG2] How did it come about that you gave up / did not take on this activity? | What motivated you [at the time / to start]?  What expectations did you have of your activity?  Did the barriers or constraints you named make participation more difficult?  [BeG2] Were the difficulties the reason you gave up your voluntary activity?  [BeG3] What role did your health situation play? |
| [BeG1 & BeG3] What does your voluntary activity mean to you — for your everyday life and your well-being?  [BeG2] What has changed since you gave up your voluntary activity?  (15 min) | Impact & meaning  Social contacts  Daily structure and tasks | Did your [BeG 2: previous] activity also give rise to contacts with younger or older people?  What has been changed [BeG 2: or lost] since? | Ask situationally about retirement or loss of employment if not raised spontaneously.  Adapt individually to the participant's biographical and health context. |
| [BeG1 & BeG3] What would you like to see happen in the area where you're involved?  [BeG2] What would need to happen for you to volunteer again — there or elsewhere?  (10–15 min) | Round in which everyone contributes | What conditions — personal, structural, or social — would make this possible or easier? | What support would be helpful? |
| Closing  (5 min) | Summary  Anything forgotten?  Any questions? | Is there anything you feel was important that we have not (sufficiently) addressed?  Is there anything else you would like to add? | Summarise key themes briefly and invite correction or addition.  Thank participants. |

**Case vignette 1.2**

Anna (34) has been volunteering for about two years at a community centre that offers homework help for children from socially disadvantaged families. She initially got involved because she wanted to "do something meaningful" and use her skills as an educator outside of her job. The feeling of being needed and being able to provide concrete help was particularly important to her. At first, she found the volunteering very enriching: the children looked forward to seeing her, small learning successes motivated her, and she also got to know other volunteers with whom a sense of community quickly developed.

Over time, however, her situation changed. The demands of her main job increased, while at the same time, more and more children were registering at the community centre without any additional volunteers. Anna increasingly felt that too much was falling on her shoulders. The organization sometimes seemed chaotic: agreements were unclear, and there was little feedback or support due to a change in the full-time coordinator.

Tensions also arose within the team, for example, when tasks were unevenly distributed or there were differing ideas about how to interact with the children. Anna is also sometimes burdened by the problems that some of the children bring with them from their families. She notices that she is often exhausted and feels pressured, as if she "can't just cancel" because she knows the kids need her. At the same time, she realizes that her own social life is suffering.

What do you think about that?

**Case vignette 1.1**

Achim (65) has been retired for a year. After many years as a tradesman, he initially looked forward to his free time, but soon realized that he was missing a sense of purpose and structure in his daily life. Therefore, he decided to volunteer and contacted a local initiative that organizes repair cafés. He liked the idea: he could share his knowledge, make connections, and make a lasting contribution.

Achim's first experiences were mostly positive. He chatted with others and was happy when a broken appliance was repaired. At the same time, however, he felt uncertain because many things were organized differently than in his former professional life. There was no clear training, responsibilities were unclear, and technical equipment or spare parts were sometimes unavailable.

After a few weeks, the situation became increasingly uncomfortable for him. He felt like he wasn't properly involved. Since the café was often run by just one person, he hardly got to know the other volunteers. At the same time, he had the impression that the others were friends, while he found it difficult to connect to them.

What do you think about that?

# **Focus Group Guide — BeG 1–3 (part 2)**

| Introduction:  At our last meeting, we discussed volunteering and its significance to you. Today, we'd like to build on that by linking volunteering with a new topic: We're interested in whether voluntary engagement and, more broadly, social participation can be supported by general practice. There's a concept for this (Social Prescribing), primarily in Great Britain, the USA, and Canada, where it's already being practiced. Social Prescribing (SP) offers general practitioners the opportunity to issue a kind of prescription for "social activity"—which can be very diverse, ranging from self-help groups and sports classes to arts and cultural offerings or even voluntary activities. Because volunteering was highlighted as so important in our first funding phase, we've explicitly included it again, even though SP is much broader in scope and aims to provide access to far more accessible opportunities. General practitioners refer patients with a social prescription to so-called link workers – trained individuals or social workers – who then work with the patients to determine which services or activities might be suitable for them.  In Germany, SP is currently the subject of much discussion, especially given the scarcity of resources in the healthcare system, demographic changes, and loneliness among the elderly. Social Prescribing aims to prevent loneliness and address social risk factors. The fact that the topic is currently receiving considerable attention is also evident in the film excerpt we will show shortly.  Film: Unedited: 7 min; shortened: 4 min: <https://www.3sat.de/kultur/kulturzeit/museum-auf-rezept-100.html>  The film presents a very positive example, but our goal is to develop a comprehensive picture, including both opportunities and obstacles/problems. We would now like to discuss this with you using a few case studies. If you can think of anything that goes beyond the examples, feel free to mention it (either from your own experience or from theoretical considerations).  (10-15 min) | | | |
| --- | --- | --- | --- |
| Narrative prompt | **Check // Memos** | **Specific questions** | **Directive and maintaining questions** |
| Prior experience with GP referrals to social or health activities | Cooking courses  Sports groups  Self-help groups  Counselling services  Arts and cultural offers | Have you ever been referred by a GP or other health professional to a social activity or community offer? | How did you experience that? Was it helpful? |
| Case vignette 2.1: Retirement  (20 min) | Finding the right activity  Potential difficulties | What are your thoughts on this example?  Where do you see potential difficulties or risks?  What type of voluntary activity might be suitable for him? | Would you accept the GP's offer if you were in Ralf's position?  What would need to be in place for this to work well?  What do you think would actually help Ralf? |
| Case vignette 2.2: Chronic illness  (20 min) | Impact of health condition on voluntary activity | What are your thoughts on this example?  What potential difficulties or challenges do you see?  What type of voluntary activity might be a good fit for her? | Would you take up the offer, if you were in Martina's position?  Do you think Martina could manage volunteering alongside her illness?  Do you think she would take it up? |
| Case vignette 2.3: Volunteering in a care home  (if time allows, 20 min) | Confronting one's own fears  Giving to others to receive for oneself  Risk of overload | What are your thoughts on this example?  What opportunities or difficulties do you see with this approach? | Do you think Gabriele would be motivated to take up volunteering in this context?  Would you follow the suggestion if you were in Gabriele's position?  Do you think this could reduce Gabriele's fear of becoming care-dependent?  How do you feel about the GP making this kind of suggestion? |
| Closing  (5 min) | Summary  Anything forgotten?  Any questions? | Is there anything you feel was important that we have not (sufficiently) addressed?  Is there anything else you would like to add? | Summarise key themes briefly and invite correction or addition.  Thank participants. |

**Case vignette 2.2 - Chronic Illness**

Martina, 52 years old, worked as an administrative clerk before her illness. She was reliable and enjoyed interacting with her colleagues and the structured nature of her work.

Eight years ago, Martina was diagnosed with a combination of chronic back pain and depressive episodes. Despite outpatient treatments and adjustments to her job, her condition worsened. A year ago, she was finally classified as permanently and fully unable to work. Since then, she has been receiving a disability pension.

Martina has been living alone since her youngest son moved out six months ago. She has been separated from the father of her two sons for many years. Her social contacts have dwindled over the years, and her self-esteem has also suffered. On “good days,” she can be active for a few hours; on “bad days,” she stays in bed. She describes herself as “no longer able to cope with the demands of the job market,” yet at the same time she doesn’t want to “be useless.” As part of an earlier rehabilitation follow-up program, a social worker brought up the topic of voluntary engagement. Martina showed interest—she wants to get involved and could also really use the expense allowance that many volunteer positions offer. However, she also has serious concerns: “What if I have to lift something there? What if I have to cancel? Will I be a burden again?”

What do you think about this?

**Case vignette 2.1 - Retirement**

Ralf (aged 65) used to be a safety engineer and has been retired for a year. He really enjoyed his first few months of retirement; at last he had time to paint his garage, prune the trees in the garden and repair the old shelving in the cellar. His wife Antje is still working as a nursery teacher and used to enjoy being able to read the newspaper in peace after work until he came home. Now she sometimes gets a bit irritated when he wants to have a chat with her.

Ralf is one of the first among his friends and acquaintances to have retired. Only one close friend has also retired, but he’s so tied up with family commitments that they don’t meet as often as they used to. During the day (when Antje is at work), he’s getting increasingly bored. In recent weeks, he’s started watching a lot of television and occasionally even opens a beer at lunchtime. Because he always seems listless and tired, Antje has suggested that he might like to see the GP. The GP believes he is suffering from mild depression, which would improve with more social contact, a regular daily routine and set tasks. He advises him to make an appointment at a counselling centre for voluntary activities.

What do you think about this?

**Case vignette 2.3 - Volunteering in a nursing home**

Gabriele (aged 70) has been living alone in her flat since her husband’s death a year ago. Apart from a few minor ailments, she is in good health, but she is now reaching an age at which her own parents once needed support. Following her father’s death, she and her sister looked after their mother, who was in increasing need of care. This meant her mother was able to die at home without ever having to move into a care home. As her own daughter has been living in Vienna for 15 years and has started a family there, the prospect of a care home is increasingly on her mind. She doesn’t know much about care homes and finds it hard to imagine what life is like there. At her last appointment, Gabriele raised the subject with her GP and asked if she could provide her with more information about care homes. Her doctor suggested she try volunteering at a care home to get a feel for everyday life there. Volunteers at care homes are usually involved in leisure activities, such as going for walks, reading aloud or running small errands.

What do you think about this?

**Focus Group Guide — FachG**

| Introduction:  This project is a follow-up to our previous project, “Medicine in old age”. In the first funding phase, we also conducted focus group discussions with doctors, nursing staff and people over 75 years of age. Among other things, these showed that social participation and engagement are important factors for well-being and health in later life. Remaining socially active, feeling included, and staying physically and mentally fit were repeatedly emphasized as important for a good later life. Volunteering was repeatedly mentioned as a good way to achieve this.  We are now taking up these aspects in the second funding phase: we want to find out to what extent the so-called Social Prescribing (SP) approach offers an opportunity for GPs to support social participation and perhaps even social or voluntary activities. Social Prescribing tries to use the authority of doctors, or of medical prescriptions, to motivate people to take part in different activities. GPs can then issue a kind of prescription for a “social activity” — which can vary from a self-help group or sports class to arts and cultural activities, or even volunteering. Because volunteering was highlighted as so important in our first funding phase, we have included it explicitly again, even though SP is much broader and aims to refer people to much lower-threshold opportunities as well.  The concept already exists in Great Britain, the USA and Canada. It is also being widely discussed in Germany at the moment, especially against the backdrop of limited resources in the health system, demographic change and loneliness in older age. Social Prescribing is intended to help prevent loneliness and address social risk factors. The fact that the topic is currently being somewhat “pushed” is also reflected in the film clip we are about to show. It presents a very positive example, although our aim is to develop a more comprehensive picture, including both opportunities and barriers/problems.  Film: Unedited: 7 min; shortened: 4 min: <https://www.3sat.de/kultur/kulturzeit/museum-auf-rezept-100.html> | | | |
| --- | --- | --- | --- |
| Narrative prompt | **Check // Memos** | **Specific questions** | **Directive and maintaining questions** |
| What comes to mind when you watch the film? How does it make you feel? | regarding the case in the film  Social Prescribing  the specific activity (art) | What are your previous experiences with referring people to social or health-related activities?  Can you give some examples? | What feelings does the film evoke in you? |
| We will now show a graphic (Figure 1) presenting the tasks associated with each role: (1) GPs identify need and potentially suitable people and refer them to (2) link workers (social work), who work with the patient to identify the most suitable form of activity, and then refer them to (3) volunteering coordinators, who integrate patients into their own structures. | | | |
| How do you view your respective scope of duties and your role in Social Prescribing? | What do GPs think about identifying need?  What do social workers have to say about this?  What do volunteer coordinators think about having people with specific needs referred to them? | How can you imagine interdisciplinary collaboration with the other professional groups? | Where do you see problems?  What possibilities do you see?  How feasible do you think the concept is?  Do you think the concept will be accepted? |
| Who do you think Social Prescribing is suitable for? | Own previous experiences | For which people is SP less suitable?  How, in your view, can people suitable for SP be identified?  How open do you think your patients/clients are to SP?  What barriers and obstacles do you think are likely? |  |
| Please tell us about your experiences of loneliness in later life in your professional environment. | (Perceived) frequency of loneliness  Reasons for loneliness  Coping strategies by those affected and by professionals | What do you observe among your patients/clients who are engaged in voluntary or social activities? Do you notice differences compared with those who are not engaged?  Do you notice differences among patients/clients/volunteers when they begin to get involved? | How do you deal with loneliness in practice, in the advice centre, or as a volunteering coordinator? |
| Closing | Summary  Forgotten anything?  Any questions? | listed questions  Have we forgotten anything that is important to you?  Is there anything else you would like to add that we have not talked about enough? | |

Figure 1: Possible Roles and Responsibilities in Social Prescribing
